# Characterization of joining copper rods by upset technique and investigating the effect of preheat treatment on the weld characteristics

**DOI:** 10.1038/s41598-025-12707-y

**Published:** 2025-07-29

**Authors:** M. Essam El-Rafey, Ali El-Ashram, Ismael M. Albadrany, Eslam Syala

**Affiliations:** 1 Department of Materials Science, Institute of Graduate Studies and Researches (IGSR), Alexandria University, 163 Horreya Avenue, Shatby, 21526 Alexandria Egypt; 2 Department of Production Engineering, Faculty of Engineering, Alexandria University, Horreya Avenue, Shatby, 21544 Alexandria Egypt; 3 Al-Saqlawiyah District Directorate, Al-Saqlawiyah, 31012 Anbar Province Republic of Iraq

**Keywords:** Copper, Upset welding, Electrical conductivity, Recrystallization, Engineering, Materials science, Characterization and analytical techniques

## Abstract

The present work studies the rod-rupture problem that occurs in the Copper and Mechanical State Company during the upcast production of 8 mm copper rods. This rod-breaking problem arises at the time of rolling the upcasted coils due to reasons of electricity fluctuations, impurities in the metal, etc. Upset welding, as a non-melting joining, was chosen and investigated to weld the copper rods to produce coils with the predetermined length for commercial applications. The welding solution was applied to avoid the negative effects of returning the cut drawn length back to the start of the production line to draw the predetermined length of the coil as one unit, which consumes extensive time and cost. The welding was performed by altering the applied welding current in three modes to investigate the effect of changing the heat input on the properties of the joints. Alternatively, the impact of preheat treatment of the welded joints on the various studied properties was also studied. The joint quality of the resultant welded rods has been characterized by studying their mechanical properties (in terms of tensile stress and elongation%), electrical conductivity (by more than one measuring method), metallographic examination, as well as morphological characterization. The results revealed that the tensile stress, average elongation%, as well as electrical conductivity related to the welded rods, were acceptable concerning the unwelded base metal, even in the case of a higher input heat. Moreover, a smooth and crack-free microscopic structure of the welded drawn wires was obtained, despite the nonperformance of preheat treatment, revealing that the heat treatment has no notable impact on the studied properties.

## Introduction

Electronic systems and their electrotechnical elements, such as wire conductors, other parts, and cables, are developed fields where there is a need for new materials that allow for a speedy, lossless transmission of electric signals. In particular, copper is the main element that is widely used in electrical and electronic devices because of its excellent electrical and thermal conductivities, which may be ascribed to its face-centered cubic (FCC) structure. This grain structure makes copper strong, formable, resistant to both corrosion and fatigue, and has a low overall cost compared to silver, which recommends its use. Also, it is recyclable and can be remelted and reused many times without depletion of its physical, electrical, mechanical, or chemical properties; only its pure form can be used for electrical wires and cables, and can also be exposed to cold working due to its formability and ductility^1,2^. Generally, electrical wires are produced through the upcasting technology, which is designed for direct processing via drawing wires and microwires from rods. These rods are sometimes exposed to rupture during casting, which represents an obstacle to continuous production. Defects entrapped within the copper, such as cracks and hydrogen gas inclusion, are the main causes of this rupture^3^. At this time, there are two solutions to address this problem. The first is taking the produced length of the coil and returning it again to the beginning of the production line, and this solution is costly and time-consuming. The second solution is to repair the rod in the cutting zone by welding while maintaining that all of its properties are the same. Welding is the fabrication process that joins two dissimilar metals or two pieces of the same metal with or without the use of filler metal. The process must maintain both the original mechanical and electrical properties of the welded rod. There are several methods, such as shielded metal arc, oxy-fuel gas, laser beam, electron beam, ultrasonic, resistance spot, brazing, or soldering, in which welding can be made. Some of these methods use filler materials, while others, such as Thomson electric welding, do not use extraneous materials such as filler rods, shields, or fluxes^4,5^. Upset welding is one of the preferred ways to weld copper, which is a heat-resistance welding technique with pressure in the contact area. Where it is a non-fusion welding mode and occurs in the solid state, the metal at the welding joint is heated by current resistance to a temperature where recrystallization can rapidly occur. At the same time, a pressure force is applied to join the contacted upset metal. The microstructure in the welded area may be affected depending on the temperature at which the pressure force is applied^6^. In this method of welding, there are several factors to be considered in the welding area formed between the pieces of metal. These factors involve the amount of current, the duration of the welding time, and the force in the joining area with the thickness of the metal^7^. Hashimoto et al. studied the preheat treatment for the welding of copper by nickel plating. They found that the pre-heat treatment for the base metal was beneficial in avoiding corrosion and eliminating any unwanted inclusions that may be found at the dissimilar metals’ contact area^8^. M. Shamsian et al. successfully welded both aluminum and copper narrow rods using upset resistance welding. They studied the welding parameters and found that increasing the welding current increased the thickness of the reaction layer and decreased the required upset force, which in turn enhanced the tensile strength, hardness, and electrical resistance at the joints than those of the base metals. The in-situ heat treatment after the welding was found to have a slight impact on the increase in the reaction layers’ thickness. They found that the whole welding process had no notable influence on the electrical conductivity of the weld zone^7^. S. Marimuthu et al. successfully joined Monel (K-500) with Copper (ETP) by the friction welding mechanism for boiler heat exchanger purposes. The results showed an increase in the tensile strength with an amplification in the upset pressure up to 30 bar. The tensile strength of the weld joint increased by increasing the friction time up to 7 s. The yield strength of the welding area increased by 33% compared to the base metal. Micro and macro surface morphology examination revealed good metallurgical bonding of the welding interface zone^9^. Łukasz Morawiński, et al. succeeded in welding the copper rods by applying the rotary friction welding. They used the conical contact surface geometry with a slant angle of 8° for the intended full contact between the welded surfaces. Concerning the mechanical properties, they found that both the microhardness and the tensile strength decreased by ≈ 10% in the joints compared to the base metal^10^. Generally and although the advantages of the upset welding method, such as the lack of need for filler metal, the removal of any found impurities from the weld, the reduction of the zone affected by heat to a minimum, the variety of applications and reliability, it also has some drawbacks such as the need for joint preparation (smoothing and cleaning), the balance of heat between the two workpieces and special designed equipment’s are required^7,11^. In this study, an industrial issue manifested in the rupture of the copper coil (rod) during the drawing from the production line was discussed, analyzed, and solved. The traditional solution in the factory was returning the drawn length back to the start of the production line to draw the predetermined length of the coil as one unit, which consumes extensive time and cost. This study applied upset welding to achieve the targeted length of the coil and also discussed the effect of preheat treatment of the joints to be welded and its impact on the studied characteristics. Alternatively, mechanical, electrical, and structural properties of the welding area were studied to ensure that they are identical to the base metal and the produced coil is as a single piece.

## Experimental work (procedures, characterization)

### Procedures

#### Samples

The copper rods samples were brought from Copper and Mechanical State Company, Iraq, (where the coil rupture problem during the upcasting occurs) with a diameter of 8 mm ф and lengths of 250 and 800 mm, whose chemical composition as indicated in Table 1 using the quantum spectrometry analysis technique. The analysis exhibits the purity of the copper rods, that is 99.99%, which refers to the 4 N grade according to the International Annealing Copper Standard (IACS). The rest of the elements, such as Ag, Al, As, etc., have no significant effect on the chemical purity or other properties where they are specified as trace elements. The specimens were cleaned of any rust, dust, oxides, or other contamination before welding by applying pickling paste, and then the two opposite faces of each sample were flattened.

**Table 1:** The chemical analysis of the copper specimens.

Element	(ppm)
Ag	9.30
Al	0.39
As	0.89
Bi	0.10
Cd	0.10
Co	0.10
Cr	0.46
Fe	4.56
Mg	0.10
Mn	0.66
Ni	1.11
P	1.14
Pb	0.10
S	0.10
Sb	2.80
Se	0.10
Si	0.10
Sn	2.92
Te	0.10
Zn	2.52
Cu^*^	99.99(%) ^*^

#### Welding process

**Fig. 1:**
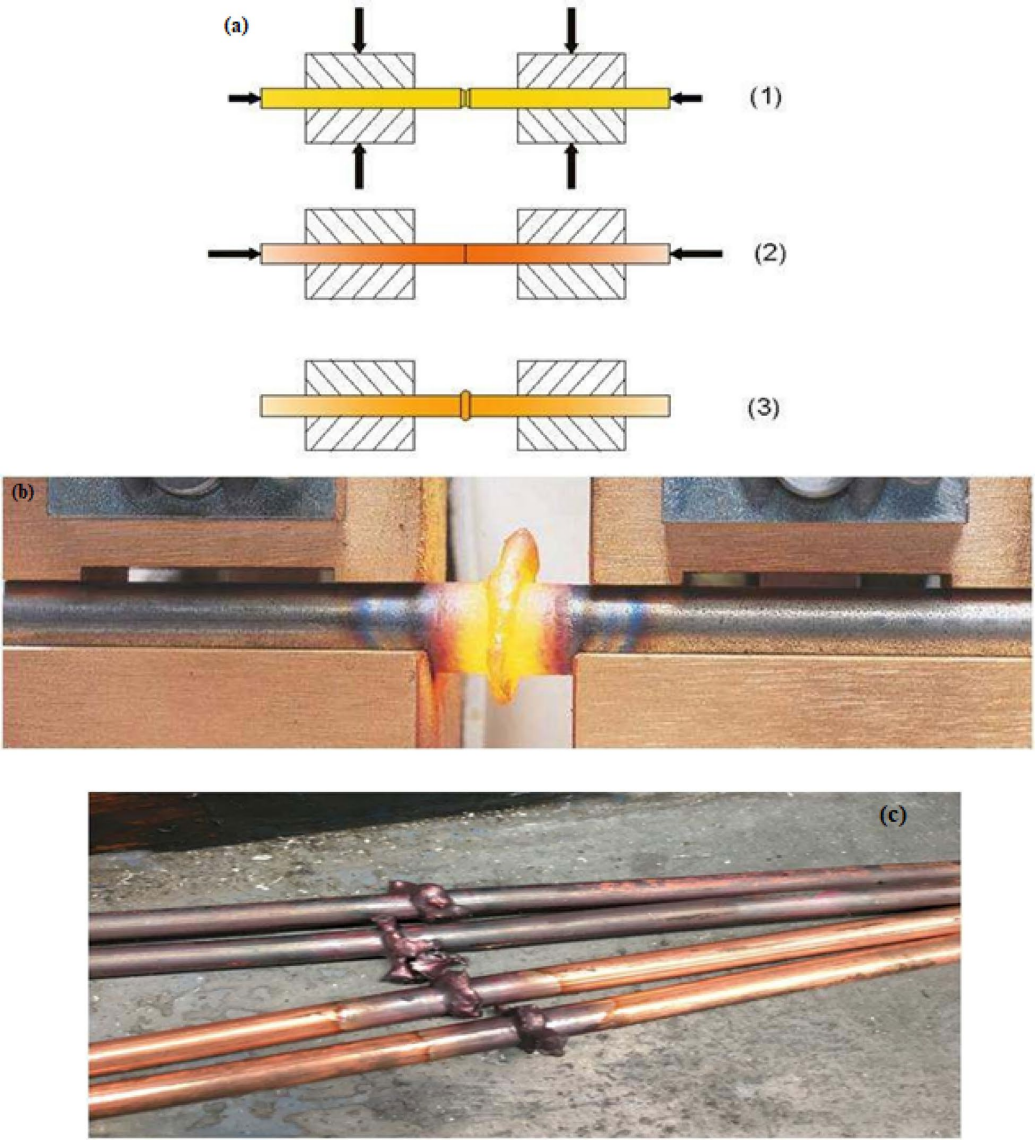
(**a**) Graphical representation of upset welding stages, (**b**) the real implemented welding process, and (**c**) some of the real welded copper specimens.

The samples were upset-welded by clamping the two metal objects to be welded together in separate electrode jaws in a butted position. When the two pieces touch together by remaining under low pressure, a gradient dense electrical current progresses from one piece to the other, and while the current is flowing, the electrical resistance heats the faces until the recrystallization temperature. At that recrystallization stage, a high-pressure force is applied to the joint from one side to complete the welding action. Thereafter, the electrical current is switched off then the pressure force is released. The representation of the process and some of the welded specimens are displayed in Fig. 1. The welding was performed by a German August Strecker electrical resistance machine Type: 2 A; 50 Hz frequency and 6.2 kA operating current at a duty cycle of 15%. The experimental welding parameters as well as the category of sample groups are presented in detail in Table 2. Eighteen samples were prepared for welding. Six of those samples were welded using high heat energy (Q6) mode. The other six samples were welded using medium heat energy (Q5); while the last six samples were welded using the low heat energy position (Q4) mode. Three of the six samples in each group have been preheated before welding. The heat-treating has been achieved by applying a low current that is passed through the welding area to rise the temperature less than the recrystallization temperature of the metal, and this was applied before allowing the passage of a heavy current. All welded samples were 8 ф × 250 mm for each piece.

**Table 2:** The applied working parameters and input heat of the welding machine.

Welding mode and coding	Voltage (V)	Current (A)	Power (W)	Heat input (J)
High heat input group (Q6)	2.6	143.49	373.1	746.14
Medium heat input group (Q5)	2.2	121.44	267.19	534.22
Low heat input group (Q4)	1.8	99.34	178.81	357.62

### Characterization

#### The mechanical testing

##### Tensile test

The tensile testing has been carried out to study the dominance of welding variables on the mechanical characteristics of the weld zone compared to those of the unwelded rod. The test was carried out according to ASTM B33 requirements, using the Universal Electromechanical Testing Machine, China, with a force capacity of 50 KN. All tested samples were 8 Φ × 250 mm, where the welding area was in the middle of the length of the tested samples. The test was carried out at ambient room temperature.

##### Drawing test

**Fig. 2:**
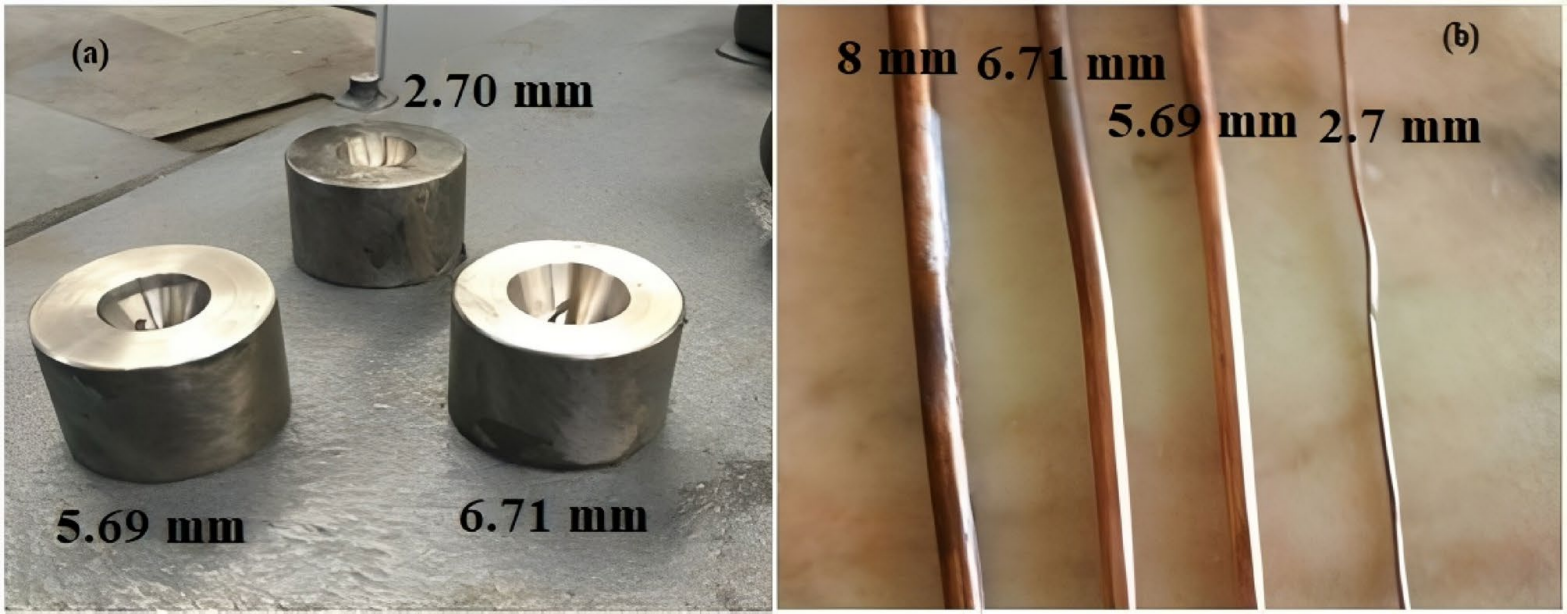
(**a**) SPD drawing dies, and (**b**) samples of the drawn copper specimens.

The drawing test was performed to investigate the formability of the welding area. The test was applied to the samples of the three modes (i.e., Q4, Q5, and Q6) to compare the outputs of the three positions. The test procedures started with passing the copper rods between rotational pointing rolls to reduce the specimens’ diameters to facilitate passing the rods through the drawing dies. After that, the suitable die required to provide the predetermined diameter was located in its position on the drawing machine, and the rods were passed through it while the ends of the rods were tightened on the drawing drum. By starting the drum rotation, the rods will be forced to enter through the dies and the drawing will be accomplished. The Krollmann point rolling machine, Germany, was used in this drawing test with synthetic polycrystalline diamond (SPD) dies that have inert diameters of 6.71, 5.69, and 2.70 mm ‘respectively’ with reduction ratios of 16, 29, and 66% of the original 8 mm drawn rod. The SPD dies, accompanied by the drawn specimens with various inert diameters, are exhibited in Fig. 2.

### The electrical properties

#### The conductivity by the device

The electrical conductivity was measured at the welded joint of each rod to determine the effect of the electrical current flow in the contact area of all rods, which was also compared with the unwelded rod to ensure the quality of the weld and which of the specimens had the highest quality. 8 Φ × 800 mm samples were used to gauge the electrical conductivity after cleaning from any contamination. The measurement was carried out using a high precision resistomat type 2304, Germany, which measures the electrical resistance of the sample. The two jaws of the device clamped the specimen, and then the resistance reading was detected in microohms through the device’s monitor. Equation no.1 (according to ASTM B193) has been applied for resistivity measurements.

1 $$\:\text{r}\text{}\text{=}\frac{\text{R*W}}{\text{L}}$$

where r is the resistivity, R is the resistance in ohms, W is the weight of the sample in grams, and L is the length in meters. Subsequently, the conductivity of each sample was measured by using the conductivity measurement equation^12^.

2 $$\:\text{conductivity}\text{}\text{(IACS)\%\:=}\frac{\text{15.328}}{\text{r}}$$

#### Conductivity by eddy’s current test

It is another method for measuring the electrical conductivity of copper and confirming the measured obtained values on the basis of converting the complex impedance of the tested sample to electrical conductivity. The measurement was performed using a portable Sigmatest 2.069 instrument operating at higher frequencies up to 960 kHz. This higher frequency allows for the possible performance of accurate electrical conductivity measurements, in terms of resistivity, on thinner test pieces. The electrical conductivity value is indicated on the instrument’s LCD and the measurement was carried out at 1.5 mm away from the surface of the specimens to determine if there are any welding defects and to ensure that there is no electrical conductivity buffer with an absolute accuracy of ± 0.5% and a resolution of ± 0.1 of the measured value.

#### Metallographic characterization

This test was performed to detect the fraction of copper grains as per ASTM standards. The preparation of the specimens was performed following the procedures in^13^. First, the copper specimens were machined on their full longitudinal axis, then they were ground following the steps listed in Table 3. After that, the samples were polished according to the steps mentioned in Table 4. The etchant solution was prepared by mixing 100 − 120 ml of water or ethanol with 20 − 50 ml of hydrochloric acid (30 − 34%) with 5–10 g of ferric chloride (III) (hydrated ferric chloride, FeCl_3_6H_2_O). Mettler AE 420, a digitally sensitive balance type, was used to weigh material amounts with a readability of 0.1 mg. Finally, an Axiovert 25 CA microscope equipped with a digital camera was used to visualize the crystal’s distributions, orientations, and grain size.

**Table 3:** Grinding steps of the specimen’s Preparation for the metallographic test.

Step	PG	FG 1	FG 2	FG 3
Surface	SiC- Paper	SiC- Paper	SiC- Paper	SiC- Paper
Grit	320	800	1200	4000
Lubricant	water	Water	water	Water
rpm	300	300	300	300
Force (N)	150	150	150	150
Time (min)	As needed	1	1	1

**Table 4:** Polishing steps of the copper specimens for the metallographic test.

Step	Surface	Suspension	rpm	Force (*N*)	Time (min)
DP Coating Prep Polish	MD-Mol	DiaPro Mol	150	150	4
OP Suspensions	OP-Chem	Iron (III) nitrate	150	90	1

#### Morphological characterization

The microstructure morphology, as well as the elemental analysis, were investigated utilizing both scanning electron microscope (SEM) and energy dispersive X-ray (EDX) techniques. The characterization was performed using a JEOL-JSM-5300 series scanning microscope. Both tests were carried out by cutting the scanned specimens from the welding zone in the longitudinal direction, polishing them with a polishing paper, and cleaning them with an alcohol solution to get more information about the microstructure of the welding zones through 1000x magnification power pictures.

## Results and discussion

### The impact of power on the welding process

**Fig. 3:**
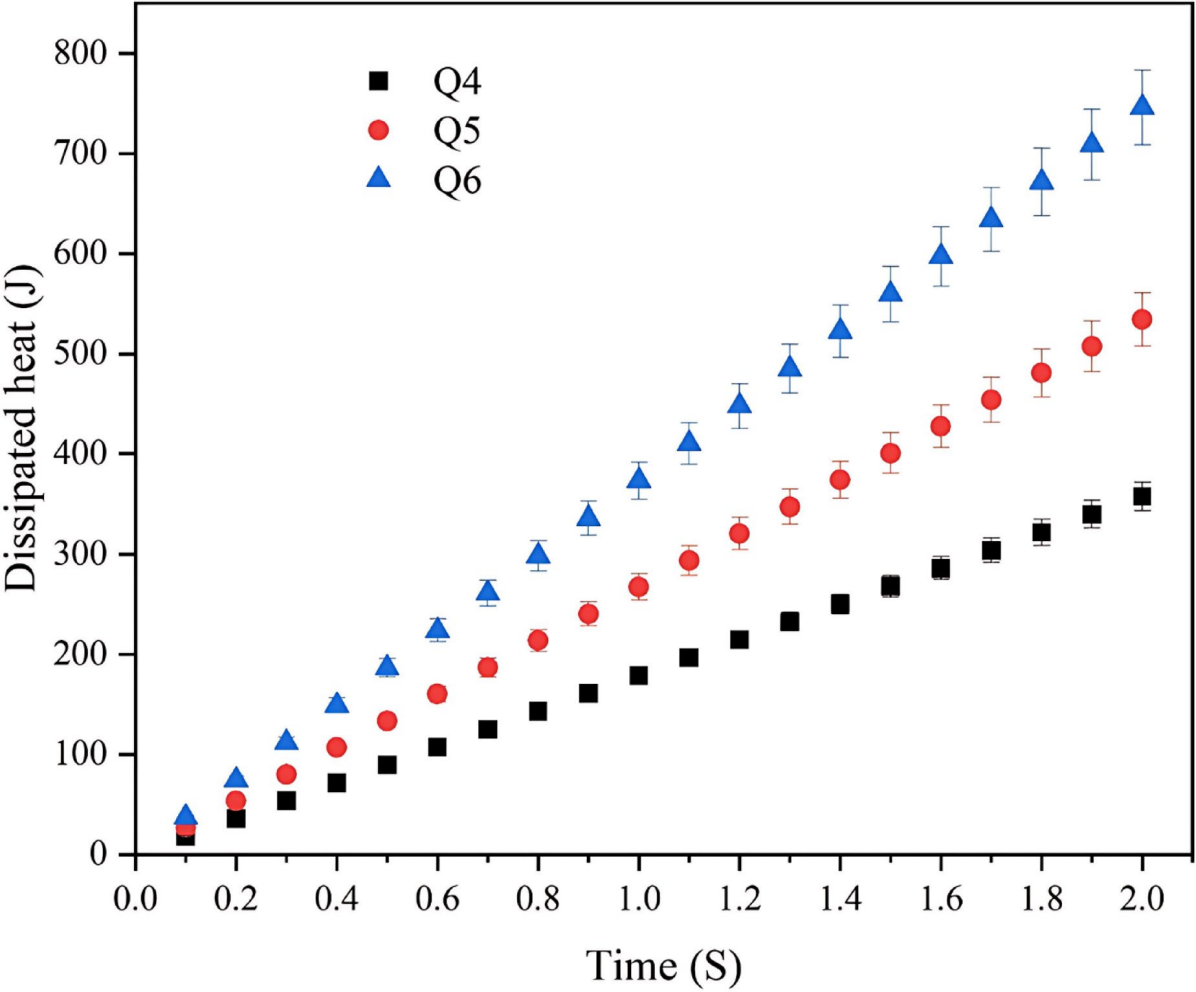
Welding heat input as a function of time with the used voltage for each welding position.

Generally, weld characteristics are considered as a function of welding time, welding power, applied pressure, and the heat dissipated in the weld for each welding position of the machine. In this method, both time and pressure were kept constant, and only changing the welding power was studied. By fixing 2s of welding time and determining the heat (energy) dissipated in the welding and altering the welding power from 178.81 (1.8 V) and 267.11 (2.2 V) to 373.07 kW (2.6 V), the Q6 welding position gave the higher energy at the same time (2s), as revealed in both Fig. 3; Table 5. In general, the maximum temperature that was achieved during the welding process was ≤ 0.4 of the melting temperature (*T*_*m*_) of the copper metal to maintain the microstructure and avoid its change.

**Table 5:** Variation of input welding heat (J) as a function of time for the different welding modes.

Time (s)	Welding mode		
	Q4	Q5	Q6
	Dissipated heat (J)		
0.1	17.88	26.71	37.31
0.2	35.76	53.42	74.61
0.3	53.64	80.13	111.92
0.4	71.52	106.84	149.23
0.5	89.40	133.55	186.53
0.6	107.28	160.26	223.84
0.7	125.17	186.98	261.15
0.8	143.05	213.69	298.45
0.9	160.93	240.40	335.76
1	178.81	267.11	373.07
1.1	196.69	293.82	410.38
1.2	214.57	320.53	447.68
1.3	232.45	347.24	484.99
1.4	250.33	373.95	522.30
1.5	268.21	400.66	559.60
1.6	286.09	427.37	596.91
1.7	303.97	454.08	634.22
1.8	321.85	480.79	671.52
1.9	339.74	507.51	708.83
2	357.62	534.22	746.14

### The mechanical properties

#### Tensile test

Table 6 shows a comparison of the mechanical properties of the welded copper samples at different welding modes. Sample No.1 refers to the unwelded sample, which has the highest elongation% and tensile stress. Samples of 2 to 4 were welded at ambient temperature without preheating, while samples of 5 to 7 were preheated at 500°C. Samples 8–10 were welded at normal temperature without preheating, with welding heat induced of 534.22 Joules, while samples 11–13 were preheated at 500 °C. Samples 14 to 16 were welded at room temperature without preheating, with welding heat induced of 357.62 Joules, while samples 17 to 19 were also preheated at 500 °C. There is no significant effect observed on the average of welding positions 6, 5, and 4 ‘respectively’ with or without preheating as a function of the welding heat dissipation (Q), as noticed in Table 6. Elongation in position 6 without preheating is 34.66% with a tensile stress of 220.60 MPa (considering the average values). For position 5, there is a decrease in elongation, which is equal to 31.66% with a tensile stress of 219.52 MPa. The commencement for position 4 without preheating shows a greater decrease in elongation up to 20.85% and the tensile stress is 203.37 MPa. The inhomogeneity of particles in the welding zone decreases the mechanical properties, which occurs as a repercussion of low welding energy. For the specimens of the welding positions 6, 5, and 4 ‘respectively’ that were preheated, there is a noticeable decrease between the average values of the specimens of the 6 and 4 positions by ≈ 20 MPa in the tensile strength and ≈ 11% in the elongation. The annealing process in the welding zone caused an increase in elongation and a decrease in the tensile strength as a result of the preheating process. The heat treatment process caused both the grains and the links between the atoms to align, causing them to consume more tensile strength with the annealing process and vice versa.

**Table 6:** The mechanical testing results.

Sp. No	Sp. symbol	Welding mode	Input Heat (J)	Tensile stress (MPa)	Average tensile stress (MPa)	Elongation%	Average Elongation %
1	A0*	Control	---	232.809	232.809	40	40
2	A_1_	Q6	746.14	219.856	220.602	35	34.66
3	A_2_			218.345		33.5	
4	A_3_			223.604		35.5	
5	B_1_	Q6 preheated	746.14	220.173	217.709	36.2	36.1
6	B_2_			219.531		35.48	
7	B_3_			213.425		36.62	
8	C_1_	Q5	534.22	216.586	219.521	31.48	31.66
9	C_2_			222.584		32	
10	C_3_			219.393		31.5	
11	D_1_	Q5 preheated	534.22	211.123	216.823	34.1	33.42
12	D_2_			220.692		32.5	
13	D_3_			218.654		33.66	
14	E_1_	Q4	357.62	204.005	203.371	21.63	20.85
15	E_2_			202.533		20.01	
16	E_3_			203.575		20.91	
17	F_1_	Q4 preheated	357.62	200.120	199.228	23.5	25.23
18	F_2_			198.875		26.22	
19	F_3_			198.689		25.97	

^*^A_0_ refers to the unwelded specimen.

#### The drawing test

**Fig. 4:**
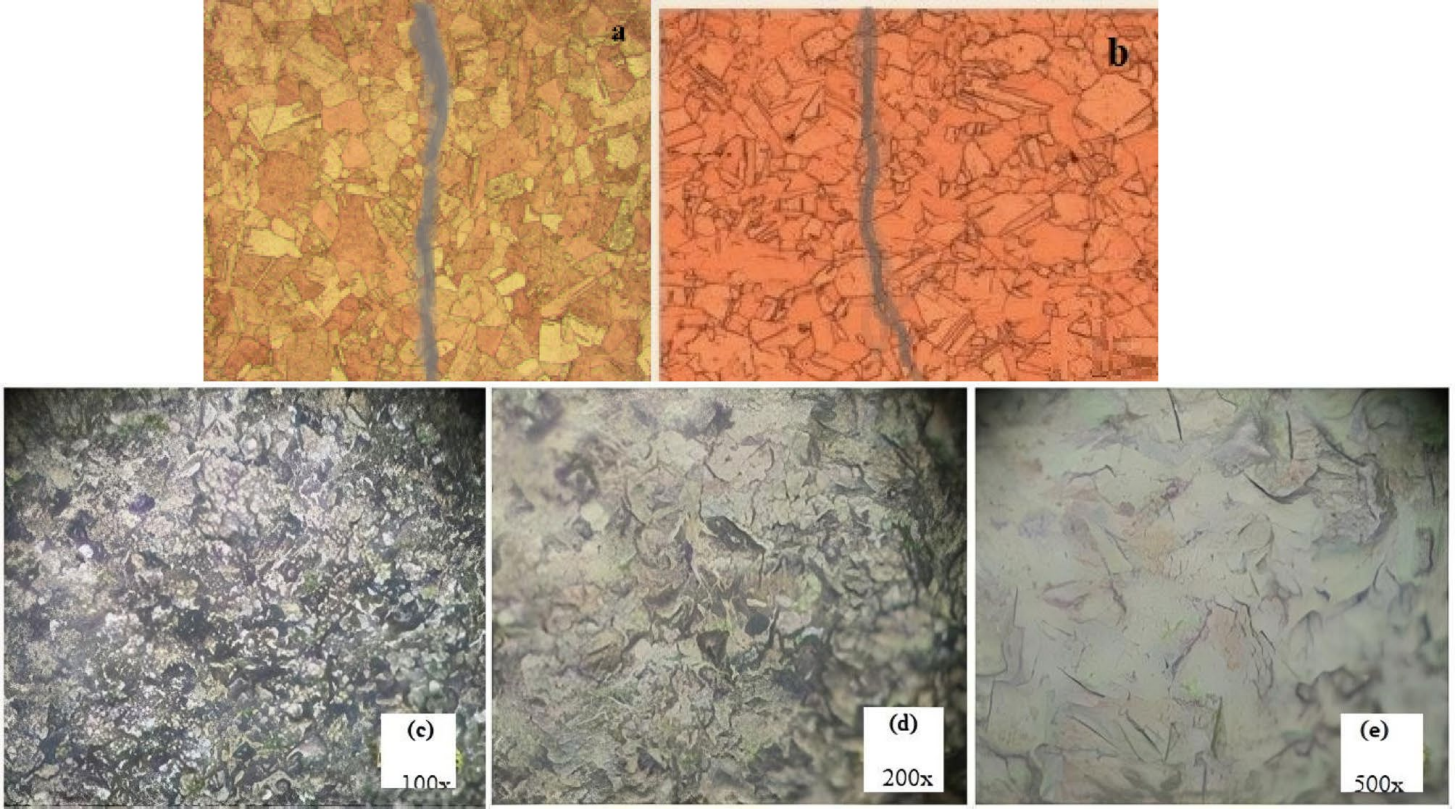
Metallographic images of the specimens welded by( **a**) Q4,( **b**) Q5 welding modes, and( **c-e)** Q6 mode at different magnifications.

In this measurement, nine specimens, classified into three groups representing the three welding positions (i.e., Q4, Q5, and Q6), were tested. The samples representing the Q4 and Q5 positions failed in the drawing test due to the crack that appeared in the longitudinal image at a diameter of 4.7 mm, as revealed in Fig. 4a, b. The samples welded according to the Q6 welding input position offered good results by decreasing the diameter from 8 mm to 6.71, 5.69, and 4.70 mm ‘respectively’ without revealing any defects by reducing the diameters. This is confirmed in Fig. 4 (c-e), which exhibits no cracks for the specimens welded by the Q6 position, contrary to those welded following the Q4 and Q5 positions. This appearance can be explained on the basis that the high-power input utilized in the welding operation at the Q6 position generated enough induced heat for recrystallization. The recrystallization occurrence led to a more homogeneous structure^14,15^ as displayed in Fig. 4c-e.

### The electrical properties

#### The conductivity by the resistomat device

This section will discuss the conductivity measurement that was achieved using the Resistomat 2304 apparatus. Comparisons have been made between the conductivity properties of the base unwelded metal and those of the welds with various heat induced in the welding process. The specimens were cut to guarantee that the cross-sectional area of the weld was exactly in the middle length of the sample. Table 7, which displays the electrical conductivity values, shows that the copper sample without welding exhibits the highest conductivity with a value of 101.3 compared to the welded samples. The samples in this test were classified in the same manner as in the mechanical properties section, concerning the coding, welding mode, and heat treatment conditions. It is presented that position 6 (Q6) showed an electrical conductivity of 100.74 without preheating and 100.55% IACS with preheating. Position 5 offered a conductivity of 100.44 without preheating and 100.38% IACS with preheating. Position 4 gave an electrical conductivity of 99.02 without preheating and 98.15% IACS with preheating. Electrical conductivity gradually decreased with the decrease of the heat-induced in the welding zone due to an increase in the count of grain boundaries (grain boundary relaxation), where there is insufficient time for grain growth, which represents an obstacle for the current flow in the contact area^16,17^. The increase in the number of grain boundaries occurs based on the recrystallization of particles, which represents the main aspect of the hot formation of the upsetting process. Similarly, the heat treatment offered a neutral/negative impact on the electrical conductivity, where it offered a chance for recrystallization of atoms that allowed increasing the number of grain boundaries.

**Table 7:** The electrical properties testing results by both the resistomat device and the eddy’s current method.

Sp. no	Sp. symbol	Welding mode	Electrical conductivity by Resistomat (IACS)	Average conductivity (IACS)	Conductivity applying Eddy’s current method (MS/m)	
					Measuring from the ends	Measuring from the welding area
1	A0	Control	101.30	101.30	59	59
2	A_1_	Q6	100.77	100.74	59	58
3	A_2_		100.75			
4	A_3_		100.68			
5	B_1_	Q6 preheated	100.48	100.55	59	57
6	B_2_		100.34			
7	B_3_		100.50			
8	C_1_	Q5	100.53	100.44	59	57
9	C_2_		100.63			
10	C_3_		100.47			
11	D_1_	Q5 preheated	100.33	100.38	59	57.5
12	D_2_		100.37			
13	D_3_		100.42			
14	E_1_	Q4	100.01	99.02	59	55
15	E_2_		98.87			
16	E_3_		98.18			
17	F_1_	Q4 preheated	99.04	98.15	59	54.5
18	F_2_		98.25			
19	F_3_		97.16			

#### The conductivity by eddy’s current instrument

In this section, electrical conductivity was tested by using the Sigmatest 2.069 device to confirm the trend of the obtained measurements revealed by the Resistomat 2304 apparatus. The first part of the test was performed at the unwelded ends of the copper rods, providing the results of 59 MS/m (100% IACS). As revealed in Table 7, the conductivity also decreases with decreasing heat induced in the welding zone through the three welding positions. The average conductivity of position Q6 showed 58 MS/m without preheating and 57 MS/m with preheating performing; similarly, position Q5 presented 57 MS/m conductivity without preheating and 57.5 MS/m with preheating, while Q4 revealed 55 MS/m conductivity without preheating and 54.5 MS/m in the preheating zone. Compared to other positions, welding at higher energy (Q6 position without preheating) showed optimal welding conditions related to unwelded copper. This can be explained based on the non-existence of welding defects because of the complete fusion by the action of the higher energy utilized in the welding process^18^and this can be considered as a non-destructive testing (NDT) for the obtained welding zone. Preheated specimens offer lower electrical conductivity than the un-preheated ones, which may be due to the precipitation of the solid solution atoms inside the matrix and the single-phase microstructure changes that increase the electrons scattering effect and hence reduce the electrical conductivity^19,20^. Comparing the results of both methods (Table 7) revealed that they generally offered the same trend for the conductivity, and they confirmed each other.

#### Metallographic characterization

**Fig. 5:**
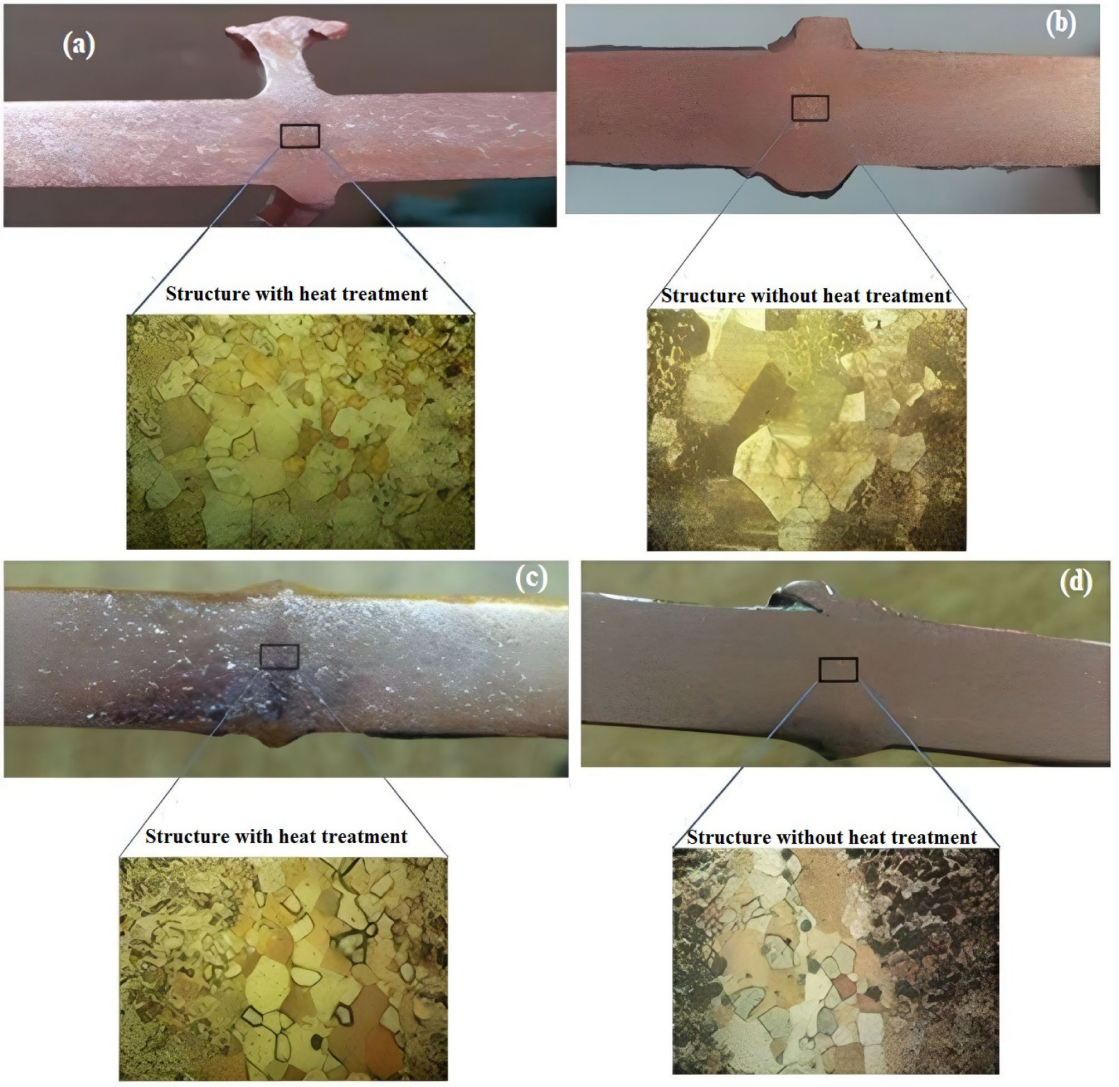
Microscopic images for Q6 (**a**,** b**) and Q5 (**c**,** d**) welding modes specimens with and without heat treatment, respectively.

Based on the estimation of the total number in the image and then dividing by the total area of the image, which is equal to 12 in², the result will be the number of particles per square inch at the magnification power (100 x) according to the ASTM E562 standard method^21^. Calculating the grain size by applying the method mentioned above shows that the grain size for the control sample (base metal without welding) is 36.1 Gr./ in² at 100x magnification power. The grain sizes for the Q6, Q5 and Q4 welding positions without and with preheating are 24, 29, 22, 28, 21, and 23.5 Gr./ in², respectively. It is found from the data that increasing welding power (voltage and current) increased the input welding heat and hence increased the grain number per unit area. On the other side, the grain growth rate in positions 6, 5, and 4 increased with heat treatment is 80, 78, and 65% ‘respectively’ as can be observed in Fig. 5a-d. The variation in the grain size of the metal was due to the annealing process that took place during the welding process. The annealing process caused secondary grain growth, which affected the final grain size of the metal^22^.

#### The morphological characterization

##### SEM and EDX analyses

**Fig. 6:**
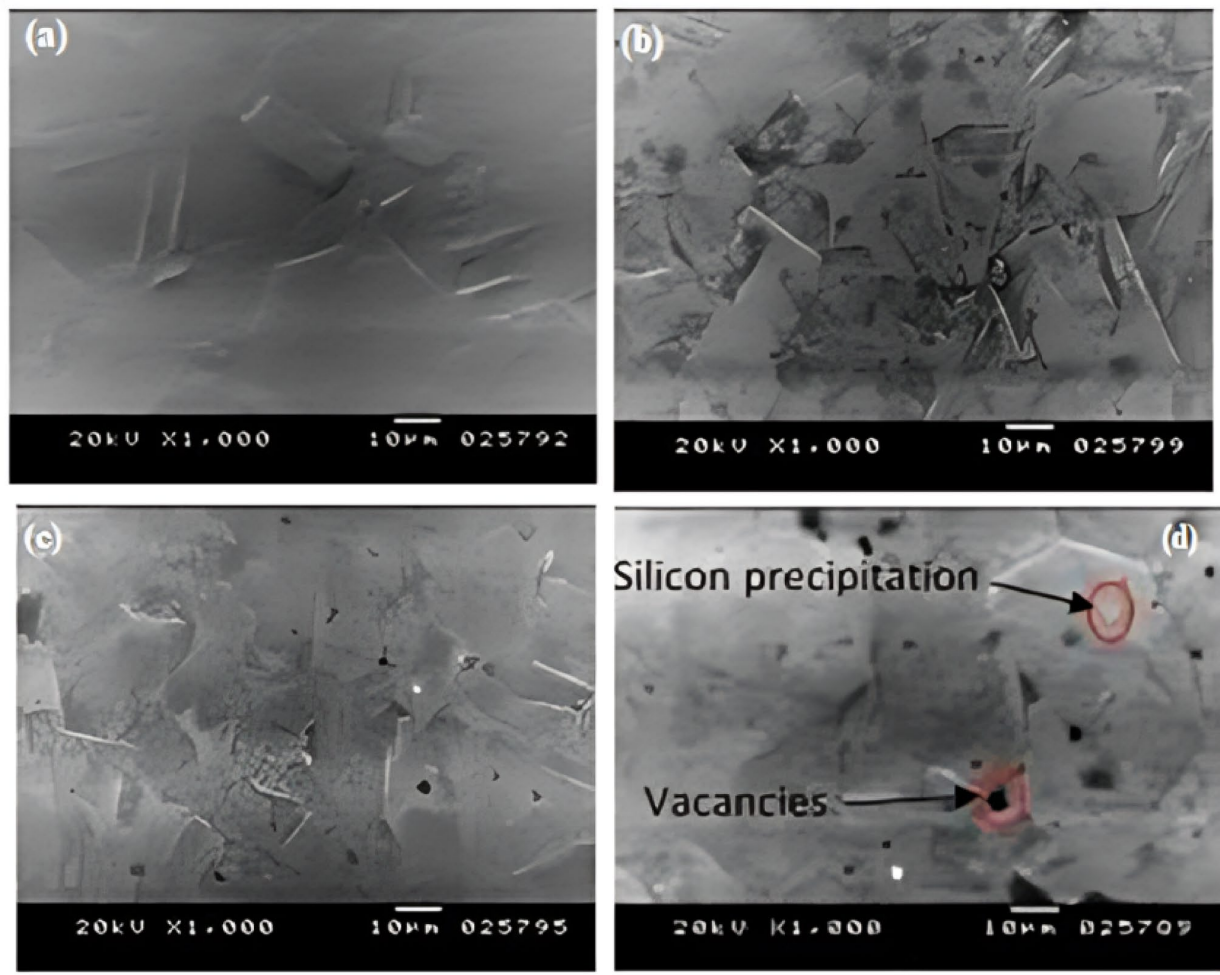
SEM images for Q6 (**a**,** b**) and Q5 (**c**,** d**) welding modes specimens without and with heat treatment, respectively.

**Fig. 7:**
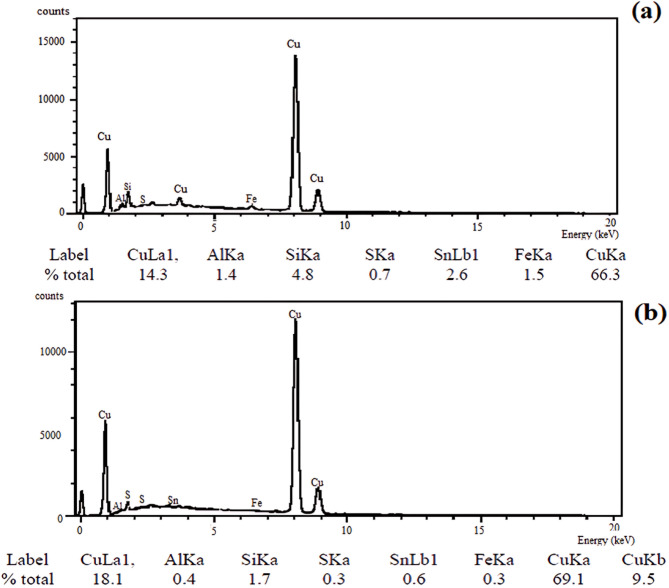
EDX spectroscopy results for copper specimens welded by (**a**) Q5, and (**b**) Q6 welding modes with preheating process.

In these tests, the unheated-treated samples exhibited a relatively smooth and uniform structure, without entrapped oxides or voids, as shown in Fig. 6a,c. On the contrary, the dark spots, contaminants, and voids in the preheated specimens, as seen in Fig. 6b,d are the results of the heat treatment process, which increases the chances for the origination of these inclusions within the structure^23^. The energy-dispersive X-ray was used to detect any impurities or contaminations in the microstructure of the specimen. EDX analysis in Fig. 7a, b showed that the dark spots in the SEM photos of the preheated specimens are vacancies that occurred upon the metal solidification. The other gray spots are silicon crystals that appear because of their poor solubility in the molten copper metal.

## Conclusion

This study focused on the solution to the problem of copper rod rupture during upcasting, which slows down or stops the process, consumes more time, cost, and energy due to the need to restart the casting process for the broken coil to produce it as a single length. The upset butt-welding technique was used in-line to weld the broken rod, and the weld properties were evaluated to ensure the high quality of the welded rod and that it is as one unit, free from any joints. Additionally, the influence of the heat treatment process before welding was tested and discussed. Both mechanical properties (in terms of tensile stress and elongation%) and electrical properties in terms of conductivity were the best for copper rods welded applying the high-input energy welding position (Q6). This welding position gave a relatively uniform and homogeneous structure as confirmed by SEM micrographs. Measuring the electrical conductivity by Eddy current was considered as a check by one of the NDT techniques for the quality of the welding, which showed that there are no cracks or discontinuities in welding by high-input energy position. Generally, the heat treatment revealed a neutral or slight negative impact on the investigated mechanical and electrical properties, where it offered a chance for recrystallization of atoms that allowed increasing the number of grain boundaries which in turn maintain or slightly deteriorate the studied property for the same sample when the results are compared with each other. This study is mainly of economic concerns, where there should be no obstacles to the continuous copper production, which represents the core of almost all types of electrical equipment and electrical transportation devices around us. In future studies, it is recommended to use the novel upset-welding technique that provides dual-force butt welding and evaluate the effect of this technique on the microstructure, mechanical, and electrical properties of welded rods, and compare the results with the properties of the unwelded base metal.

## Data Availability

All data generated or analyzed during this study are included in this published article.

## References

[1] Gan, B. et al. Electrical conductivity of copper under ultrahigh pressure and temperature conditions by both experiments and first-principles simulations. *Phys. Rev. B*. **109**, 115129. 10.1103/PhysRevB.109.115129 (2024).

[2] Islam, S. S. U., Khan, N. Z. & Siddiquee, A. N. Review of heat treatment of welded sheet metals during past 15 years. *Compr. Mater. Process. (Second Edition)*. **12**, 41–56. 10.1016/B978-0-323-96020-5.00046-7 (2024).

[3] Sulitsin, A. V., Mysik, R. K. & Brusnitsyn, S. V. Quality upgrade of the copper wire rod produced by combined continuous casting and rolling method. *Non-Ferrous Met.* (2), 47–51. 10.17580/nfm.2016.12.10 (2016).

[4] Emerson, J. N., Marrero-Jackson, E. H., Nemets, G. A., Okuniewski, M. A. & Wharry, J. P. Nuclear reactor pressure vessel welds: A critical and historical review of microstructures, mechanical properties, irradiation effects, and future opportunities. *Mater. Des.* **244**, 113134. 10.1016/j.matdes.2024.113134 (2024).

[5] Electric Welding. *Science*, 15, 157–58. *JSTOR*, (1890). http://www.jstor.org/stable/1763186 10.1126/science.ns-15.370.157 17753379

[6] Kerstens, N. F. H. & Richardson, I. M. Heat distribution in resistance upset butt welding. *J. Mater. Process. Technol.* **209** (5), 2715–2722. 10.1016/j.jmatprotec.2008.06.015 (2009).

[7] Shamsian, M., Movahedi, M., Kokabi, A. H. & Ozlati, A. Upset-resistance welding of aluminium to copper rods: effect of interface on performance. *Mater. Sci. Technol.* **34**, 1830–1838. 10.1080/02670836.2018.1482254 (2018).

[8] Hashimoto, K., Sato, T. & Niwa, K. Laser welding copper and copper alloys. *J. Laser Appl.* **3** (1), 21–25. 10.2351/1.4745272 (1991).

[9] Marimuthu, S., Balasubramanian, K. R. & Kannan, T. T. M. Mechanical and surface morphology study of Monel–Copper joint by rotary friction welding. *Mater. Today*. **37**, 419–424. 10.1016/j.matpr.2020.05.401 (2021).

[10] Morawiński, Ł. et al. Solid-state welding of ultrafine grained copper rods. *Arch. Civ. Mech. Eng.* **21** (3), 89. 10.1007/s43452-021-00244-0 (2021).

[11] Singh, G. & Arora, N. Design and development of micro upset welding setup. *Int. J. Mech. Eng. Rob. Res.* **2** (2), 51–58 (2013).

[12] https://www.ipc.org/sites/default/files/test_methods_docs/2.5.14a.pdf

[13] Caron, R. N., Barth, R. G. & Tyler, D. E. Metallography and microstructures of copper and its alloys. In: * Metallography and Microstructures*. 775 – 788. (ASM International (2004).

[14] Wang, Y., Wang, H., Peng, S., Xia, B. & Zhu, H. The dominant role of recrystallization and grain growth behaviors in the simulated welding Heat-Affected zone of High-Mn steel. *Materials* **17** (10), 2218. 10.3390/ma17102218 (2024). 38793285 10.3390/ma17102218 PMC11123467

[15] Dmytryk, V. V., Glushko, A. V. & Tsar, A. K. Recrystallization in the metal of welding joints of steam trucks. *Вопросы Атомной Науки И Техники*. **5** (123), 49–52 (2019).

[16] Matsui, I., Kanetake, M., Mori, H., Takigawa, Y. & Higashi, K. Relationship between grain boundary relaxation strengthening and orientation in electrodeposited bulk nanocrystalline Ni alloys. *Mater. Lett.* **205**, 211–214. 10.1016/j.matlet.2017.06.094 (2017).

[17] Li, L. C., Chai, M. Y., Li, Y. Q., Bai, W. J. & Duan, Q. Effect of welding heat input on grain size and microstructure of 316L stainless steel welded joint. *Appl. Mech. Mater.* **331**, 578–582. 10.4028/www.scientific.net/AMM.331.578 (2013).

[18] Yang, T., Liu, J., Zhuang, Y., Sun, K. & Chen, W. Studies on the formation mechanism of incomplete fusion defects in ultra-narrow gap laser wire filling welding. *Opt. Laser Technol.* **129**, 106275. 10.1016/j.optlastec.2020.106275 (2020).

[19] Cao, F., Qin, Z., Chen, C., Xu, Y. & Huang, L. Effect of heat treatment on microstructure, hardness and electrical conductivity of as-extruded ZK80 Mg alloy. *J. Phys. : Conf. Ser.* **1699** (1), 012030. 10.1088/1742-6596/1699/1/012030 (2020).

[20] Appel, J. Electron-electron scattering and transport phenomena in nonpolar semiconductors. *Phys. Rev.* **122** (6), 1760. 10.1103/PhysRev.122.1760 (1961).

[21] Bhadeshia, H. K. D. H. Introduction to quantitative metallography. *Material Sci. & Metall. Part. II Metallography* (2009).

[22] Anwar, S., Rehman, A. U., Usmani, Y. & Al-Samhan, A. M. Influence of post weld heat treatment on the grain size, and mechanical properties of the alloy-800 h rotary friction weld joints. *Materials* **14** (16), 4366. 10.3390/ma14164366 (2021). 34442888 10.3390/ma14164366 PMC8402188

[23] Guo, S., Karasev, A. V., Tilliander, A. & Jönsson, P. G. Evaluation of sulfide inclusions before and after deformation of steel by using the electrolytic extraction method. *Metals* **11** (4), 543. 10.3390/met11040543 (2021).

